# Qualitative and quantitative analysis of 18F-GP1 positron emission tomography in thrombotic cardiovascular disease

**DOI:** 10.1038/s41598-024-77151-w

**Published:** 2024-11-05

**Authors:** Beth Whittington, Evangelos Tzolos, Shruti Joshi, Rong Bing, Jack Andrews, Krithika Loganath, Neil Craig, Craig Balmforth, Laura Clark, Christophe Lucatelli, Mark G MacAskill, Adriana A. S. Tavares, Tim Clark, Nicholas L. Mills, Jennifer Nash, Damini Dey, Piotr J. Slomka, Norman Koglin, Andrew W. Stephens, Marc R. Dweck, Michelle C. Williams, William Whiteley, Edwin J. R. van Beek, Joanna M. Wardlaw, David E. Newby

**Affiliations:** 1 BHF Centre for Cardiovascular Science, University of Edinburgh, 47 Little France Crescent, Edinburgh, EH16 4TJ UK; 2 Edinburgh Imaging, Queen’s Medical Research Institute, Edinburgh, UK; 3 Centre for Clinical Brain Sciences, University of Edinburgh, Edinburgh, UK; 4 Usher Institute, University of Edinburgh, Edinburgh, UK; 5 Life Molecular Imaging GmbH, Berlin, Germany; 6 Departments of Medicine (Division of Artificial Intelligence in Medicine), Biomedical Imaging Research Institute, Cedars-Sinai Medical Centre, Los Angeles, USA; 7 UK Dementia Research Institute Centre, University of Edinburgh, Edinburgh, UK

**Keywords:** Cardiovascular diseases, Platelets, Stroke, Molecular imaging, Radionuclide imaging

## Abstract

^18^F-GP1 is a novel highly specific radiotracer that binds to activated platelets and thrombus. We aimed to establish the observer repeatability of coronary, carotid and cerebral ^18^F-GP1 uptake in patients presenting with acute myocardial infarction or ischaemic stroke. Forty-three patients presenting with acute myocardial infarction or ischaemic stroke underwent hybrid positron emission tomography (PET) and computed tomography (CT) angiography. Qualitative and quantitative assessment of ^18^F-GP1 uptake was performed on coronary arteries, carotid arteries and brain parenchyma. Qualitative uptake of ^18^F-GP1 had excellent intraobserver and interobserver agreement, with complete agreement for the presence or absence of visual ^18^F-GP1 uptake. For quantitative analysis, there were excellent intraclass correlation coefficients for intraobserver repeatability for coronary artery, carotid artery and brain parenchymal SUV_max_ and TBR_max_ measurements (all ≥ 0.92). Coronary artery and brain parenchymal analyses showed the strongest agreement in SUV_max_ values with mean biases of − 0.04 (limits of agreement − 0.21 to 0.20) and 0.02 (limits of agreement − 0.29 to 0.32) respectively. There was good interclass correlation coefficients for interobserver repeatability for coronary artery, carotid artery and brain parenchymal SUV_max_ and TBR_max_ measurements (all ≥ 0.89). The strongest interobserver agreement was seen with brain parenchymal SUV_max_ (mean SUV_max_ 1.95 ± 0.94) and TBR_max_ (mean TBR_max_ 9.55 ± 6.56) with mean biases of − 0.05 (limits of agreement − 0.37 to 0.27) and 0.04 (limits of agreement − 0.59 to 0.52) respectively. Visual qualitative and quantitative ^18^F-GP1 PET-CT image analyses provide robust and repeatable measurements of activated platelets and thrombi within the coronary arteries, carotid arteries and brain parenchyma.

**Supplementary Information:**

The online version contains supplementary material available at 10.1038/s41598-024-77151-w.

## Introduction

Thrombotic cardiovascular diseases are the leading cause of mortality worldwide. Direct visualisation of thrombus is often difficult and requires invasive techniques, such as angiography, where the presence of thrombus is often inferred from the presence of filling defects or severe stenosis. There is a need for a more specific and non-invasive method of assessing the presence of thrombus.

^18^F-GP1 is a fluorine-18 labelled positron-emitting radiotracer that has high affinity for the activated glycoprotein IIb/IIIa receptor present on activated platelets that are present in acute thrombus. ^18^F-GP1 positron emission tomography and computed tomography (PET-CT) is a recently described method of identifying the presence of thrombus within the cardiovascular system in wide range of diseases^1–5^. In particular, it is a promising non-invasive technique for the detection of coronary thrombosis in patients with acute myocardial infarction^2^ as well as cerebrovascular thrombosis in patients with ischaemic stroke and transient ischaemic attacks^1^. This has potential for understanding the role and origin of thrombosis in these major cardiovascular diseases with the potential to influence future patient management.

The repeatability of methods used to identify ^18^F-GP1 uptake within the cardiovascular system has not been established. The aim of this study was to establish the intraobserver and interobserver repeatability of this technique for detecting and quantifying coronary artery, carotid artery and brain parenchymal uptake. Because of the transient nature of thrombus formation and the influence of therapeutic interventions, we did not perform scan-rescan reproducibility.

## Methods

### Study populations

Participants were enrolled in either the In Vivo Thrombus Imaging With ^18^F-GP1, a Novel Platelet PET Radiotracer [iThrombus] study (NCT03943966)^2^ or the Origin and Role of Thromboembolism in the Pathogenesis of Ischaemic Stroke (TORPIS) study (NCT05636748). In patients with acute myocardial infarction, all participants were over the age of 40 years with recent (within 7 days) type 1 myocardial infarction^6^ and cardiac troponin concentration > 50 times the upper reference limit awaiting or having undergone inpatient coronary angiography. In patients with acute stroke, participants were greater than 18 years old and had been diagnosed with an acute ischaemic stroke as per diagnostic criteria of the American Heart and Stroke Association guidelines^7^ within 3–4 weeks of symptom onset. Exclusion criteria were impaired renal function (estimated glomerular filtration rate < 30 mL/min/1.73 m^2^), inability to give informed consent, unable to tolerate the scanner protocol, women who were pregnant or breast feeding, or had an allergy to iodinated contrast agents. Additional exclusion criteria for the stroke cohort included evidence of haemorrhagic stroke or participation in the study would result in a delay to carotid endarterectomy surgery.

These studies were approved by the Scottish Research Ethics Committee (REC reference: 18/SS/ 0163 and 21/WS/0165), the United Kingdom Administration of Radiation Substances Advisory Committee and local institutional review board. Written informed consent was obtained from all participants. The study protocols conformed to the ethical guidelines of the 1975 Declaration of Helsinki.

### PET-CT acquisition

All patients were administered a target dose of 250 MBq ^18^F-GP1 and PET-CT angiography was performed after 60 min with a hybrid 128-multislice scanner (Biograph mCT, Siemens, Medical Systems, Erlangen, Germany). In patients with myocardial infarction, this was performed with prospective electrocardiogram-gating. PET list mode acquisition was performed with a single bed position centred on the heart and was preceded by an attenuation correction CT scan. Contrast-enhanced coronary CT angiography was performed after 30 min of PET acquisition (80 mL, Iomeron 400 contrast, Bracco Suisse S.A., Switzerland) using automated dose modulation (CARE Dose4D, Siemens, Germany) and tube voltage selection (CARE kV, Siemens, Germany) with a slice thickness of 0.6 mm in mid-diastole during held expiration.

In patients with stroke, a low-dose attenuation correction computed tomography scan was followed by acquisition of positron emission tomography data using three 20-min bed positions centred on the head and neck, the thoracic aorta and the heart. This was followed by contrast-enhanced coronary CT angiography (80 mL, Iomeron 400 contrast, Bracco Suisse S.A., Switzerland) using automated dose modulation (CARE Dose4D, Siemens, Germany) and tube voltage selection (CARE kV, Siemens, Germany) with a slice thickness of 0.6 mm in mid-diastole during held expiration. A contrast-enhanced CT angiogram was then be performed of the cerebral and carotid circulation (70 mL, Iomeron 400 contrast, Bracco Suisse S.A., Switzerland). Helical computed tomography was performed with tube voltage with tube current selected automatically based on scout images.

### Analysis of 18F-GP1 uptake

**Fig. 1:**
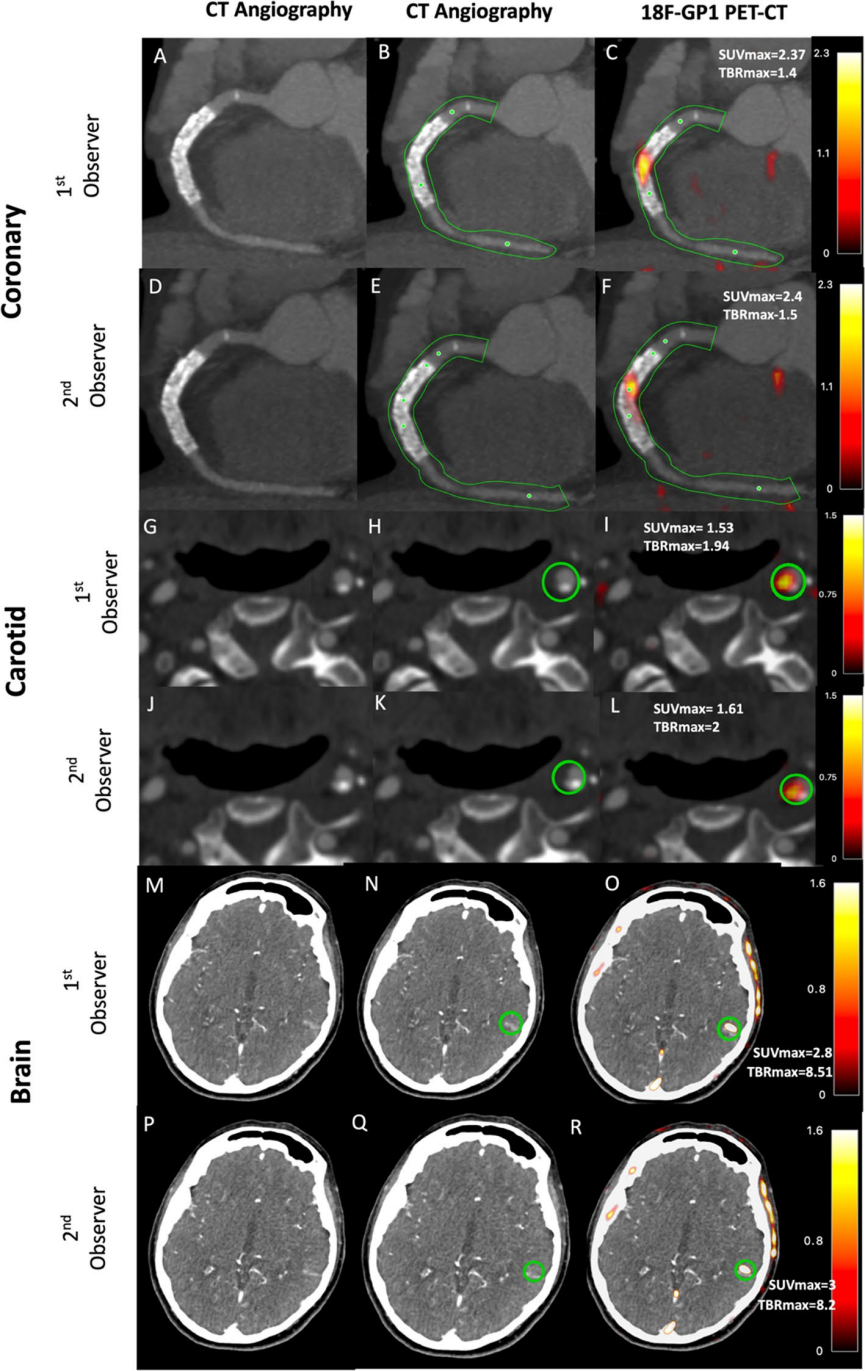
Quantitative ^18^F-GP1 positron emission tomography and computed tomography image analysis. Panels (**A**)–(**F**) show a patient presenting with inferior ST elevation myocardial infarct and receiving primary cutaneous intervention to the right coronary artery. CT angiography (**A** and **D**) with semiautomated centrelines placed to generate volumes of interest (**B** and **E**; green lines representing tubular region of interest). ^18^F-GP1 is seen within culprit right coronary artery and stented segment (**C** and **F**). Panels (**G**)–(**L**) show a patient presenting with left partial anterior circulatory stroke secondary to left carotid artery atherosclerotic disease. Carotid artery CT angiography (**G** and **J**) shows a mixed plaque disease in the internal carotid artery within the region of interest (**H** and **K**; green circle representing spherical region of interest). ^18^F-GP1 uptake seen associated with culprit plaque (**I** and **L**). Panels (**M**)–(**R**) show a patient with left partial anterior circulatory stroke with infarction seen in left temporal lobe on CT angiography (**M** and **P**) with the region on interest drawn (**N** and **Q**; green circle representing spherical region of interest). ^18^F-GP1 uptake is seen within area of infarction (**O** and **R**).

**Fig. 2:**
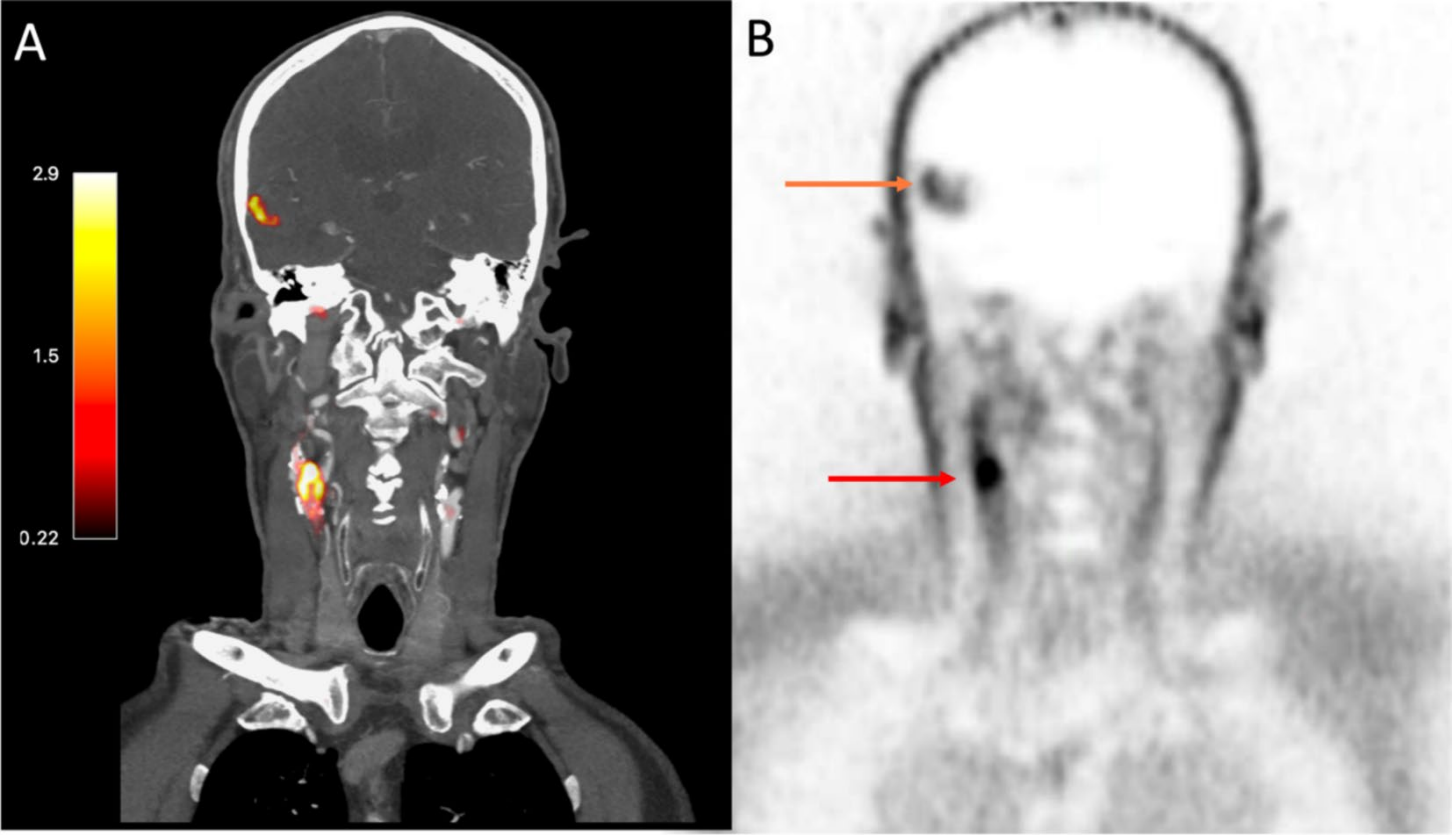
^18^F-GP1 Positron Emission Tomography images of a patient with an acute ischaemic stroke caused by large artery atherosclerosis. Images from an 81-year old gentleman presenting with headache and confusion. Panel (**A**) shows fused computed tomography and positron emission tomography images with PET uptake seen as red/orange. Corresponding PET only images in Panel (**B**). Both panels show^18^F-GP1 uptake in right carotid artery (Panel (**A**)—red/orange colour and Panel (**B**)—red arrow) indicating acute plaque rupture with thrombus embolization causing right middle cerebral artery infarct (Panel (**A**)—red/orange colour, Panel (**B**)—orange arrow).

Qualitative and quantitative assessment of ^18^F-GP1 uptake was carried out on the co-registered PET and CT angiogram images using FusionQuant software (Version v1.21.04.21, Cedars-Sinai, CA, USA) as described previously^1,8–11^. For patients with myocardial infarction, ^18^F-GP1 uptake was assessed in all three coronary arteries (left anterior descending artery, left circumflex artery and right coronary artery) where the vessel diameter was ≥ 2 mm (Fig. 1). For patients with stroke, ^18^F-GP1 uptake was assessed in the carotid arteries, the intracranial arteries and the brain parenchyma (Figs. 1 and 2). For intraobserver repeatability, the observer BW analysed consecutive scans which were then reanalysed four months later in a random order to avoid recall bias. For interobserver repeatability, SJ performed the analyses on the same sequential scans independently. Both trained observers were blinded to clinical information as well as previous analyses.

### Coronary PET-CT

#### Image analysis

For coronary ^18^F-GP1 analysis, PET images were reconstructed into 4 cardiac phases (each at 25% of the cardiac cycle) using vendor software (JS-Recon12, Siemens, Knoxville, TN, USA) as described previously^2^. PET images were reconstructed using an Ordered Subset Expectation Maximisation (OSEM) algorithm with Time of Flight (TOF) capability enabled, undergoing 4 iterations of 21 subsets and split into 4 equal gates, each representing 25% of the cardiac cycle. Reconstructions were scaled to a 512 × 512 pixel matrix and used a zoom factor of 1. Gaussian smoothing was applied with a 3-mm full-width at half-maximum kernel. Cardiac motion-corrected images were obtained from the gated PET reconstructions through PET image co-registration using a diffeomorphic registration and dedicated software (FusionQuant version 1.21.04.21, Cedars-Sinai Medical Center).

Co-registration was performed using a rigid translation of PET images using five anatomical points of reference: blood pool of left ventricle, right ventricle, great vessels and bone marrow of vertebra and sternum.

#### Coronary motion correction

To compensate for coronary artery movement, all gates were aligned to the end-diastolic position as described previously^2,11^. The first step in this process requires anatomical coronary artery data to be extracted from the CT coronary angiogram using dedicated software (FusionQuant Software, Cedars-Sinai Medical Center, Los Angeles) which applies a vessel tracking algorithm based on Bayesian maximal paths. Following this, a diffeomorphic mass-preserving image registration algorithm was applied to align the 4 gates of PET data to the end-diastolic gate. After motion correction, the 4 gates were summed together to obtain a motion-free image containing all counts from the entire PET acquisition.

#### Coronary 18F-GP1 uptake assessment

Qualitative and quantitative ^18^F-GP1 assessment was performed on the co-registered PET and CT coronary angiogram images. ^18^F-GP1 PET uptake was assessed in the left anterior descending artery, left circumflex and right coronary arteries where the vessel diameter was ≥ 2 mm. The left main coronary artery was included in the left anterior descending artery volume of interest. Uptake within the diagonal and intermediate branches of the coronary arteries (> 2 mm) were included in the left anterior descending artery whilst uptake within the obtuse marginal branches (> 2 mm) were included in the circumflex artery. Where the left circumflex or right coronary artery bifurcated, uptake was measured in the larger branch. Posterior descending artery and posterior left ventricular branches were included in the corresponding dominant vessel as described previously^2^.

For qualitative assessment, positive visual uptake was defined as discrete increased signal arising from a major epicardial coronary artery that followed its course for > 5 mm in three dimensions on orthogonal views as described previously^2,9^.

Uptake values were assessed using maximum standardised uptake values (SUV_max_), mean standardised uptake values (SUV_mean_), maximum target-to-background ratio (TBR_max_) and mean target-to-background ratio (TBR_mean_). TBR_mean_ was determined by dividing SUV_mean_ by the SUV_mean_ of the background and TBR_max_ was determined by dividing the SUV_max_ by the SUV_mean_ of the background.

SUV_mean_ and SUV_max_ values were obtained from the whole vessel as described previously (Autoplaque version 2.6, Cedars-Sinai Medical Center, Los Angeles, CA)^12^. This encompasses all the main epicardial coronary arteries and their immediate surroundings (4-mm radius) which provides a per vessel uptake assessment. We measured the SUV_max_ on the co-registered PET and coronary CT angiogram images in this tubular volume of interest. Blood pool activity was measured in the right atrium as the mean SUV in cylindrical volumes of interest (radius 10 mm and thickness 5 mm) at the level of the right coronary artery ostium^2^.

### Carotid and brain PET-CT

#### Image analysis

PET images were reconstructed using an ordered subset expectation maximisation algorithm with time of flight capability enabled, undergoing 4 iterations of 21 subsets. Reconstructions were scaled to a 512 × 512 pixel matrix and used a zoom factor of 1. Gaussian smoothing was applied with a 3-mm full-width at half-maximum kernel. Prior to analysis, reconstructed positron emission tomography images were fused with computed tomography angiograms using rigid translation of positron emission tomography images and alignment with points of reference, such as the bone marrow of the cervical vertebra and the blood pool of jugular veins and great vessels^1^.

#### Carotid artery and brain 18F-GP1 assessment

Carotid artery and brain ^18^F-GP1 uptake was assessed as described previously^1^. For qualitative assessment, carotid artery visual uptake was defined as discrete increased signal intensity within the carotid artery at the site of atherosclerotic plaque seen on all three orthogonal views and on 5 subsequent axial slices. For quantitative carotid uptake assessment, spherical volumes of interest were centred on the region of interest to give maximum standardized uptake values. Blood pool activity was determined using an average of three maximum standardized uptake values in the internal jugular vein at the level of the carotid bifurcation with sphere radius being dependent on the size of the vessel. Uptake values were assessed using SUV_max_, SUV_mean_, TBR_max_ and TBR_mean_. In the absence of visual carotid ^18^F-GP1 uptake, SUV measurements were taken at the largest atheromatous plaque within the carotid bifurcation or in the proximal 1 cm of the internal carotid artery if no plaque was present as described previously^1,13^. SUV_max_ and TBR_max_ values for visually negative and positive arteries were compared.

For qualitative assessment of the brain, positive visual uptake was defined as discrete increased signal seen on 3 orthogonal views in 5 subsequential axial slices in either the intracranial arteries or brain parenchyma. Quantitative ^18^F-GP1 brain uptake was determined using 3-dimensional spherical volumes of interest centred on the region of interest adjusted to encompass the area of visual uptake to give maximum standardized uptake values. The brain background activity was determined by placing three spherical volumes (same radius as used for uptake region of interest) over the contralateral non-infarcted brain tissue and an average of maximum standardized uptake values was taken^14,15^. Given the very low ^18^F-GP1 uptake within the brain parenchyma, the maximum standardized uptake values were used instead of the mean.

### Statistical analysis

Categorical variables were presented as numbers (percentage). Data were tested for normality of distribution using the Shapiro-Wilk test. Continuous, normally distributed variable were presented as mean ± standard deviation. Non-normally distributed continuous variables were presented as median [interquartile range]. Assessment of TBR_max_, TBR_mean_, SUV_max_ and SUV_mean_ observer repeatability were obtained using descriptive statistics with interclass correlation coefficient and Bland-Altman plots with mean bias and 95% limits of agreement. Based on 95% confidence intervals of the intraclass correlation coefficient values, < 0.5, 0.5–0.75, 0.75–0.9 and > 0.90 indicate poor, moderate, good and excellent repeatability respectively^16^. Regression models were constructed with TBR_max_ and SUV_max_ and as the independent variable and age, sex, hypertension and diabetes mellitus as the covariates. Statistical analysis was performed using the software package R (v4.0.2, R Foundation Statistical Computing, Vienna). Statistical significance was taken as two-sided *P* < 0.05.

## Results

### Study population

Participants had a mean age of 65 ± 12 years and 28% (*n* = 12/43) were female (Table 1). Patients with a recent myocardial infarction (*n* = 23) underwent PET-CT scanning a median of 7 [4–9] days from time of myocardial infarction and just over a half (*n* = 13/23) had multivessel coronary artery disease on coronary CT angiogram. All three coronary arteries were visualised for each patient resulting in 69 coronary arteries for analysis. Patients with stroke (*n* = 20) underwent PET-CT a median of 11 [9–14] days from symptom onset. The majority of patients (*n* = 19/20) had evidence of carotid atherosclerotic disease defined as the presence of calcified or non-calcified atheroma on CT angiography.

**Table 1:** Patient characteristics.

	Overall *n* = 43	Myocardial infarction *n* = 23	Stroke *n* = 20
Age (years)	65 ± 12	60 ± 8	70 ± 14
Female sex	12 (28%)	4 (17%)	8 (40%)
Body-mass index (kg/m^2^)	28 [25–31]	28 [25–31]	28 [25–35]
Current smoker	16 (37)	10 (43)	6 (30)
Ex smoker	11 (26)	8 (35)	3 (15)
Hypertension	23 (54)	8 (35)	15 (75)
Atrial fibrillation	1 (4)	0 (0)	1 (17)
Hypercholesterolaemia	19 (44)	7 (30)	12 (60)
Diabetes mellitus	6 (14)	3 (13)	3 (15)
Ischaemic heart disease	6 (14)	4 (17)	2 (10)
Peripheral vascular disease	1 (2)	1 (4)	0 (0)
Previous stroke	5 (12)	1 (4)	4 (20)
Medications			
Aspirin	34 (79)	23 (100)	11 (55)
P2Y12 receptor antagonist	37 (86.0)	23 (100)	14 (70)
Statin	43 (100)	23 (100)	20 (100)
Anticoagulation	6 (14)	0 (0)	6 (30)

Continuous variables reported as mean ± SD or median [interquartile range]; categorial variables reported as number n (%).

### Qualitative assessment presence of 18 F-GP1 uptake

There was excellent concordance for the identification of visual uptake on qualitative assessment of the coronary arteries (positive visual uptake *n* = 22/23; left anterior descending artery *n* = 6/22, right coronary artery *n* = 14/22, and left circumflex artery *n* = 2/22 ), carotid arteries (positive visual uptake *n* = 8/20) and brain (positive visual uptake *n* = 20/20) (Figs. 1 and 2). During both intraobserver and interobserver analyses, there was complete agreement for the presence or absence of visual ^18^F-GP1 uptake.

**Fig. 3:**
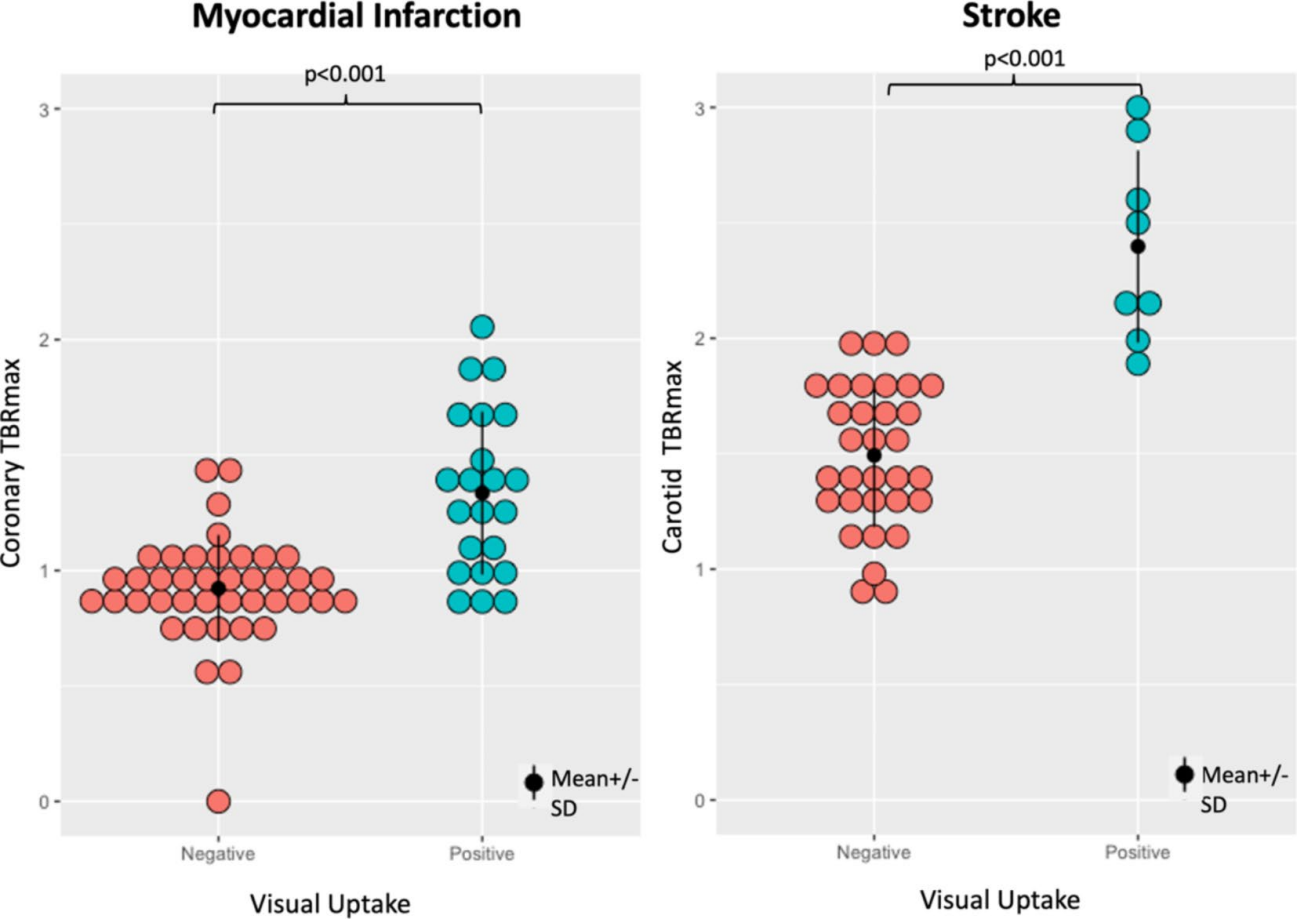
^18^F-GP1 uptake measured as maximum tissue-to-background ratio in visually assessed negative and positive coronary and carotid arteries.

As expected, higher values of SUV_max_ and TBR_max_ were demonstrated in the coronary and carotid arteries which were identified as being visually positive. For coronary arteries in the myocardial infarction cohort, the mean TBR_max_ for positive and negative qualitative assessments were 1.33 ± 0.35 and 0.9 ± 0.27 respectively. For carotid arteries in the stroke cohort, the mean TBR_max_ for positive and negative qualitative assessments were 2.40 ± 0.42 and 1.49 ± 0.31 respectively (Fig. 3).

### Quantitative assessment of 18 F-GP1 uptake

#### Intraobserver agreement

**Fig. 4:**
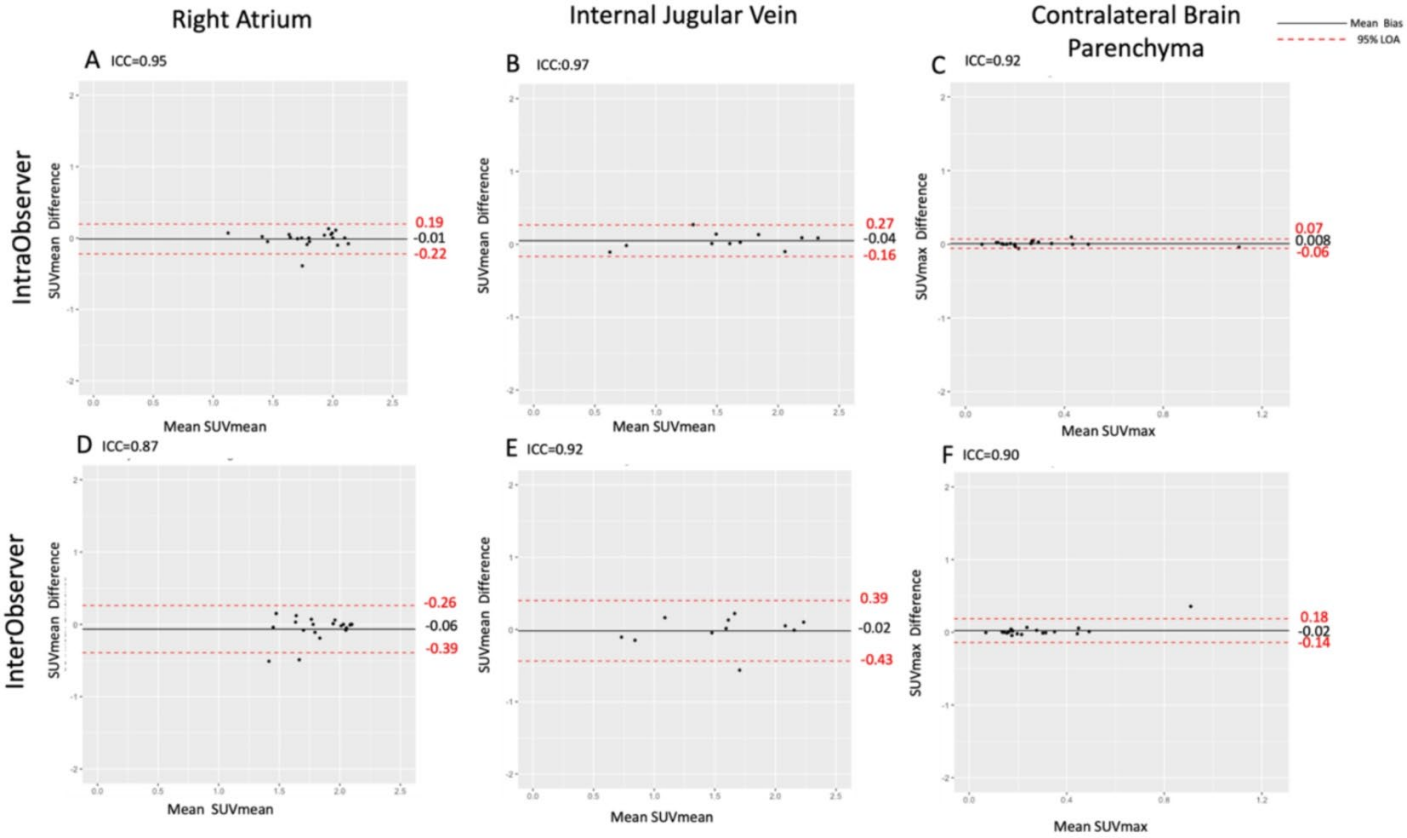
Intraobserver and interobserver agreement of background ^18^F-GP1 activity assessed for coronary arteries (first column), carotid arteries (second column) and brain (third column) uptake. For the coronary arteries, mean standardised uptake value (SUV_mean_) was measured in the right atrium; for the carotid artery, SUV_mean_ was measured in the internal jugular vein; and for the brain, SUV_max_ was measured in the unaffected contralateral brain parenchyma. *ICC* interclass correlation coefficient, *LOA* limits of agreement.

Background activity analysis showed excellent intraobserver agreement. Background activity showed no bias or difference in mean values for the right atrium (SUV_mean_ 1.88 ± 0.39, mean of differences 0.01 (limits of agreement of -0.22 to 0.19, *p* = 0.58), internal jugular vein (SUV_mean_ 1.58 ± 0.53, mean of differences 0.008, limits of agreement − 0.16 to 0.27, *p* = 0.12) and contralateral brain parenchyma (SUV_max_ 0.29 ± 0.22, mean of differences 0.04 (limits of agreement − 0.16 to 0.27, *p* = 0.28) (Fig. 4).

**Fig. 5:**
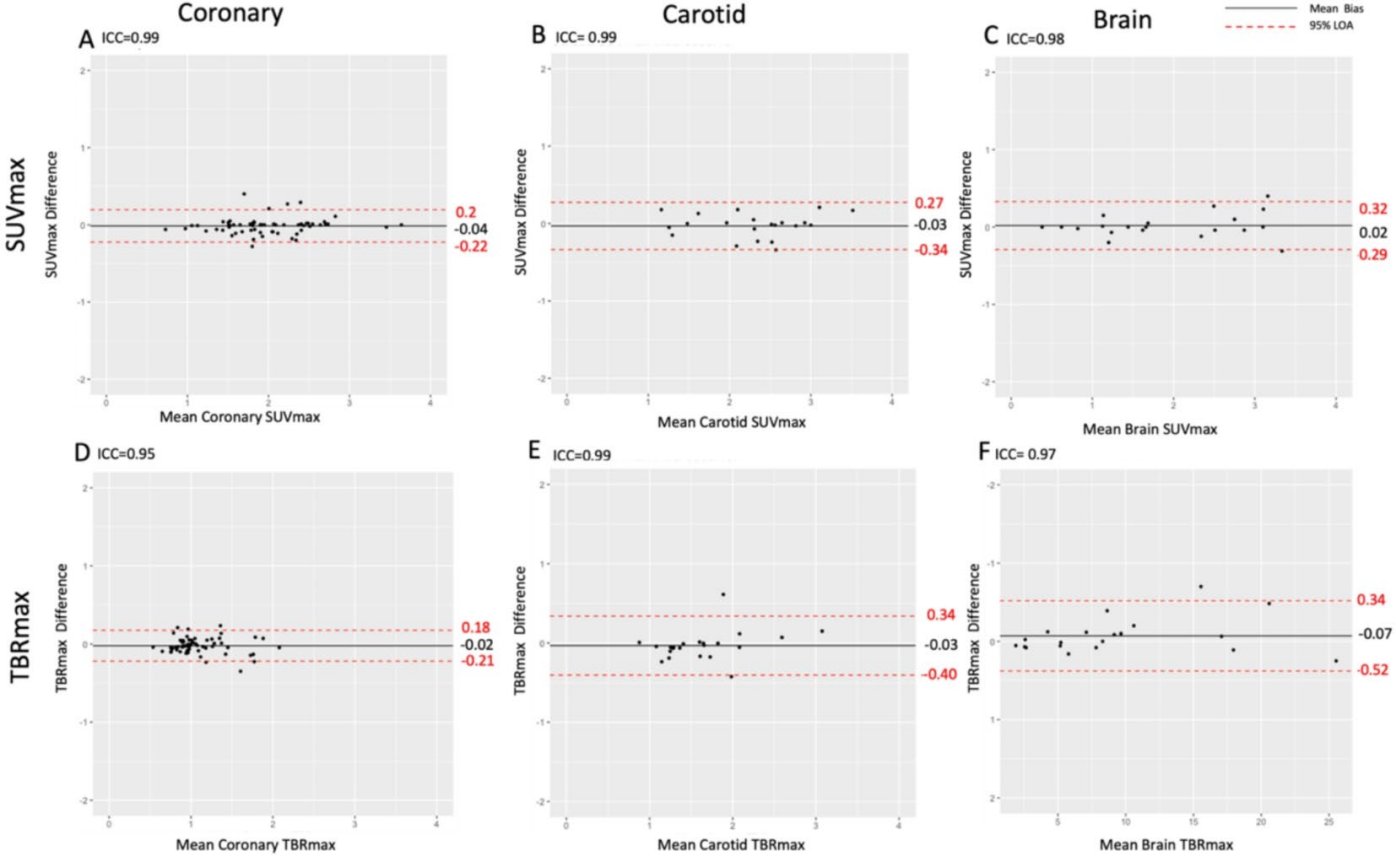
Intraobserver agreement of ^18^F-GP1 uptake in the coronary arteries (first column), carotid arteries (second column) and brain (third column) uptake. *ICC* interclass correlation coefficient, *LOA* limits of agreement. Please note the difference in x-axis scale for brain TBR_max_.

Intraobserver agreements were excellent for coronary artery, carotid arterial and brain parenchymal SUV_max_ and TBR_max_ measurements (Fig. 5; Table 2). For the coronary arteries, there were excellent agreements for both SUV_max_ and TBR_max_: intraclass correlation coefficients of 0.99 and 0.95 respectively, and mean biases of -0.04 (limits of agreement − 0.21 to 0.20) and − 0.02 (limits of agreement − 0.21 to 0.18) respectively. Similar excellent agreements were apparent for carotid artery SUV_max_ and TBR_max_: interclass correlation coefficients of 0.99 and 0.97 respectively, and mean biases of -0.03 (limits of agreement 0.34 to 0.27) and − 0.03 (limits of agreement − 0.40 to 0.34) respectively. Similar findings were also observed for brain parenchymal SUV_max_ and TBR_max_, although consistent with higher mean values, the absolute limits of agreement were wider for TBR_max_, but were proportionally small (Fig. 5; Table 2). Overall findings were similar for both SUV_mean_ and TBR_mean_ (Supplementary Figure i).

**Table 2:** Comparison of mean differences and limits of agreement in SUV_max_ and TBR_max_ in coronary, carotid and brain between intraobserver and interobserver analyses.

Cohort	Mean (± SD)	SUV_max_ Mean of differences (LOA)		*P* value
		Intraobserver	Interobserver	
Coronary	2.06 ± 0.71	– 0.04 (– 0.22 to 0.20)	0.009 (– 0.57 to 0.55)	0.89
Carotid	2.47 ± 1.07	– 0.03 (– 0.34 to 0.27)	0.01 (– 0.38 to 0.40)	0.29
Brain	1.97 ± 0.94	0.02 (– 0.29 to 0.32)	– 0.05 (– 0.37 to 0.27)	0.20

Cohort	Mean (± SD)	TBR_max_ Mean of differences (LOA)		*P* value
		Intraobserver	Interobserver	
Coronary	1.10 ± 0.35	− 0.02 (− 0.21 to 0.18)	− 0.004 (− 0.34 to 0.35)	0.35
Carotid	1.63 (± 0.52)	− 0.03 (− 0.40 to 0.34)	− 0.002 (− 0.54 to 0.54)	0.55
Brain	9.55 ± 6.56	0.07 (− 0.52 to 0.34)	0.04 (− 0.59 to 0.52)	0.70

*LOA* imits of agreement.

#### Interobserver agreement

Background activity analysis showed good interobserver agreement. As with intraobserver analysis, background activity showed no bias or difference in mean values for the right atrium (SUV_mean_ 1.90 ± 0.34, mean of differences 0.06, limits of agreement of -0.39 to 0.26, *p* = 0.07), internal jugular vein (SUV_mean_ 1.56 ± 0.50, mean of differences 0.02, limits of agreement of -0.43 and 0.39, *p* = 0.78) and contralateral brain parenchyma (SUV_max_ 0.28 ± 0.19, mean of differences − 0.02, limits of agreements − 0.14 to 0.16, *p* = 0.282) (Fig. 4).

**Fig. 6:**
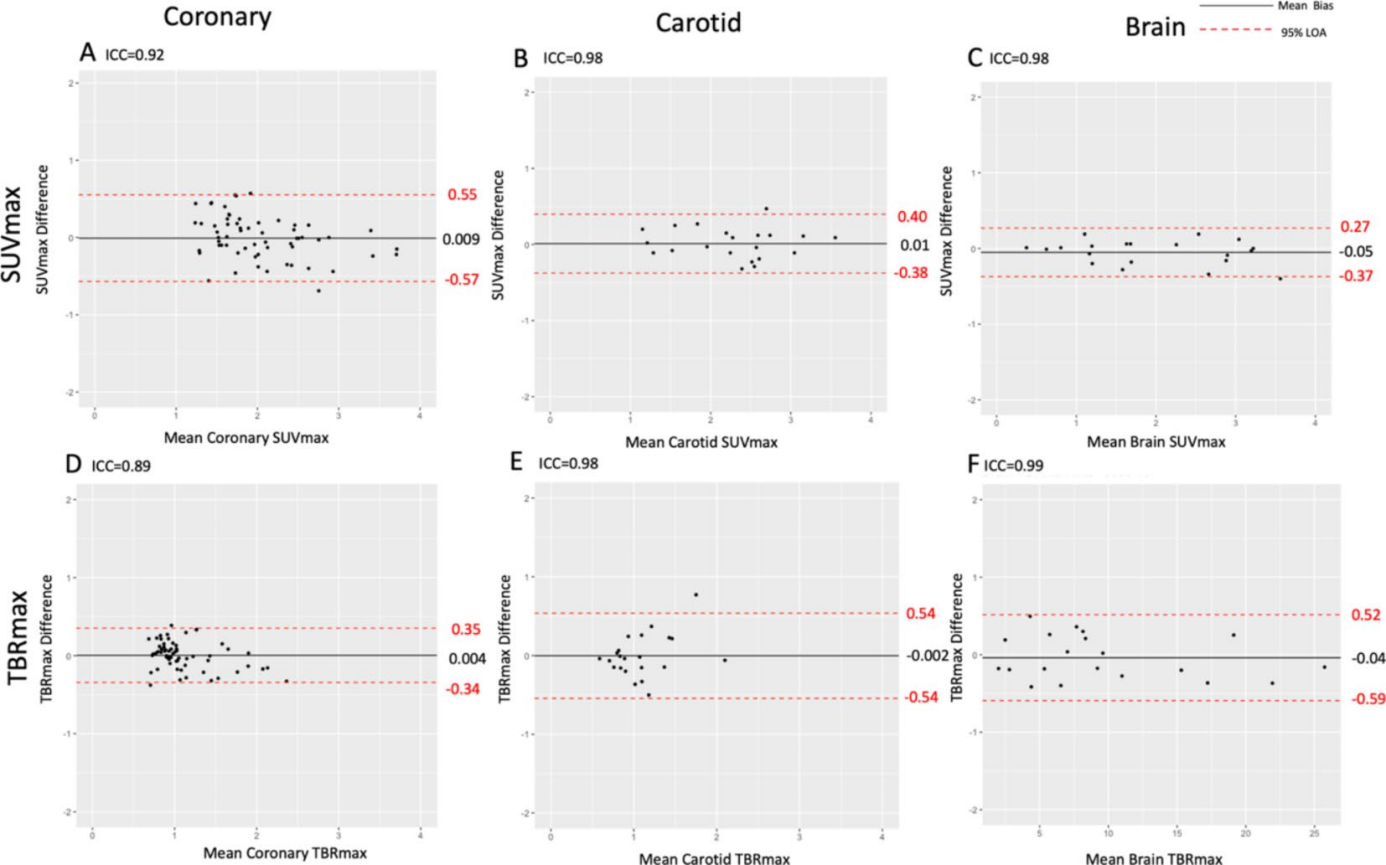
Interobserver agreement of ^18^F-GP1 uptake in the coronary arteries (first column), carotid arteries (second column) and brain (third column) uptake. *ICC* interclass correlation coefficient, *LOA* limits of agreement. Please note the difference in x-axis scale for brain TBR_max_.

The interobserver agreement was good or excellent for coronary arterial, carotid arterial and brain parenchymal SUV_max_ and TBR_max_ measurements (Fig. 6; Table 2). For the coronary arteries, SUV_max_ and TBR_max_ showed good agreement: intraclass coefficients of 0.92 and 0.89 respectively and mean biases of 0.009 (limits of agreement − 0.57 to 0.55) and 0.004 (limits of agreement − 0.34 to 0.35) respectively. Carotid artery analysis of SUV_max_ and TBR_max_ demonstrated excellent agreement: intraclass coefficients of 0.98 for both and mean biases of 0.01 (limits of agreement − 0.38 to 0.4) and − 0.002 (limits of agreement − 0.54 to 0.54) respectively. Similarly, brain parenchymal SUV_max_ and TBR_max_ analysis showed excellent agreement: intraclass coefficients of 0.98 and 0.99 respectively and mean biases of -0.05 (limits of agreement − 0.37 to 0.27) and 0.04 (limits of agreement − 0.59 to 0.52) respectively. Again, brain TBR_max_ analysis showed higher absolute mean bias values and wider limits of absolute agreement when compared to SUV_max_ values, but were proportionally smaller (Fig. 6; Table 2). These findings were similar for both SUV_mean_ and TBR_mean_ analysis (Supplementary Figure ii).

Regression models showed no effect of age, sex, hypertension and diabetes mellitus on SUV and TBR values and consequently no effect on repeatability measurements.

## Discussion

^18^F-GP1 PET is a novel non-invasive tool for in vivo imaging of activated platelets on thrombi. For the first time, we have evaluated the repeatability of qualitative and quantitative analysis of ^18^F-GP1 PET uptake in coronary arteries, carotid arteries and brain parenchyma in the context of atherothrombotic disease. We found excellent agreement in the qualitative identification of visual ^18^F-GP1 uptake in all anatomical regions, and this was further confirmed on quantitative analysis with good to excellent levels of intraobserver and interobserver repeatability. This suggests that ^18^F-GP1 PET is a robust non-invasive tool for the identification and quantification of in vivo thrombosis.

When assessing the background activity of ^18^F-GP1 PET, we used three different anatomical reference areas. For coronary analysis, the right atrium was selected for the assessment of background blood pool activity because this is an easily visualised large structure which is likely to have less variability^9,10^. Indeed, we found no evidence of bias and narrow limits of agreement for both within and between observers. For brain parenchymal analysis, we used the contralateral unaffected brain parenchyma as our background reference which is common practice for brain PET uptake studies^13–15^. Due to the blood brain barrier, there is only a very low background uptake of ^18^F-GP1 in the brain parenchyma which provides low variability in background measurements as we demonstrated in our analysis. In contrast to the right atrium and brain parenchyma, the internal jugular vein varies in size and shape, and its borders can be difficult to visualise. It also lies adjacent to the larger carotid artery which can cause spill over of PET uptake. Both the accuracy of drawing regions of interest within a small variable structure, and the potential for overspill and partial volume effects, led to greater variability and wider limits of agreement when assessing background activity, especially for between observer comparisons.

The strongest intraobserver agreements in both SUV_max_ and TBR_max_ were seen in the coronary arteries and the brain. Coronary arteries have clear separation from other vessels, and within the brain, there is very little background uptake. These factors reduce overspill of signal and facilitate clear demarcation of areas of target uptake and account for the superior repeatability of analyses in these regions. For the carotid arteries, the juxtaposition of veins and complexity of the vascular structures in the neck did lead to greater variability. This was further increased for measurements of TBR_max_ because of the greater variation in the background activity associated with the internal jugular veins. Comparing intraobserver and interobserver agreements, the mean differences of SUV_max_ and TBR_max_ values were similar for all cohorts. As anticipated, there were wider limits of agreement for the interobserver analysis because there will inevitably be differences in drawing regions of interest and interpretation of images. However, this was rather modest and overall consistent with the presence of clear and robust signals using this technique.

Interobserver quantification of brain parenchymal uptake had the strongest level of agreement for SUV_max_ whereas the coronary arteries appeared to have numerically the widest limits of agreement. The superior repeatability of brain parenchymal uptake measurements likely reflects the superior signal-to-noise ratio of brain tissue with little background activity. For the vascular structures, there is the effect of blood pool activity which will inevitably limit the discrimination of vascular wall and luminal target uptake. The coronary analysis is further complicated by a multistep analytical approach involving the drawing of centrelines of each coronary artery and motion correction. This more complex method will inevitably introduce more variability. Despite all of these challenges, we still observed excellent intraclass correlation coefficients and small mean biases for SUV_max_ and TBR_max_ in the three regions we assessed here.

We should acknowledge some limitations of our study. First, we did not perform interval scanning to derive interscan reproducibility. The is due to the effects of the passage of time and antiplatelet and anticoagulant therapy on thrombus degradation and resolution in these acute cardiovascular diseases. In a previous study, we observed potentially false negative ^18^F-GP1 uptake in culprit coronary arteries of patients with acute myocardial infarction. This was, in part, attributable to a delay in PET-CT scanning (> 7 days) from the acute presentation and there will be a reduction in thrombus burden due to antithrombotic drug interventions^2^. We do acknowledge that patients could be scanned on consecutive days but this raises both logistical challenges and additional radiation exposure. Another limitation of the study is the relatively small number of participants. Despite this, we were able to analyse 129 images for ^18^F-GP1 uptake: 69 in coronary arterial, 40 in carotid arterial and 20 brain parenchymal images. Finally, this was a single centre study and we need to explore whether this approach is generalisable by comparing images across sites, scan vendors and image analysis software packages.

There are several clinically relevant findings of our study. First, we have found good to excellent repeatability of assessing ^18^F-GP1 uptake in the coronary and carotid arteries and the brain. We have developed a standardised systematic approach to its analysis for each anatomical region and, for the coronary arteries, employed semi-automated techniques to allow for consistency in image interpretation and reporting. We conclude that this assessment is sufficiently robust for potentially clinical application. Second, we have included a high-risk population of patients, with a large proportion of multivessel coronary artery disease and bilateral carotid atherosclerosis. This population is representative of the population in which this technique is likely to be applied. Moreover, they will potentially have most to benefit from such non-invasive imaging techniques that will likely guide their management. Finally, the development of antithrombotic therapies for myocardial infarction and stroke is often hampered by a lack of non-invasive techniques to track the dynamic processes of thrombosis and thrombolysis. Other positron emission tomography techniques such as ^18^F- fluorodeoxyglucose (marker of inflammation) and ^18^F-sodium fluoride (marker of microcalcification) can identify high-risk vulnerable atherosclerotic plaque but not the thrombus resulting from an acute atherosclerotic plaque rupture. This non-invasive imaging technique has the ability to visualise active thrombus directly and holds major promise in understanding the role and origin of thrombosis in cardiovascular thrombotic disease as well as a biomarker of target engagement and treatment efficacy in trials of novel antithrombotic regimens.

In conclusion, qualitative and quantitative measurements of ^18^F-GP1 uptake on PET-CT is repeatable in patients with thrombotic cardiovascular diseases both within and between observers. This provides strong support for the use of this novel technique in ongoing clinical studies looking at the role and origin of thrombus in thrombotic cardiovascular disease which has the potential to guide the most appropriate therapy and interventions.

## New knowledge gained

The repeatability of methods used to identify ^18^F-GP1 uptake within the cardiovascular system has not previously been established. We here demonstrate that qualitative and quantitative measurements of ^18^F-GP1 PET-CT imaging in coronary arteries, carotid arteries and brain parenchyma are repeatable in cardiovascular thrombotic disorders both within and between observers. These findings support the development of these techniques use in ongoing clinical studies and potential clinical application.

## Electronic supplementary material

Below is the link to the electronic supplementary material.

Supplementary Material 1

## Data Availability

The data that support the findings of this study are available from the corresponding author upon reasonable request.
