# Supplementary material for: Qualitative and quantitative analysis of 18F-GP1 positron emission tomography in thrombotic cardiovascular disease
Source: Sci Rep. 2024 Nov 5;14:26792. doi: 10.1038/s41598-024-77151-w (PMC11538255; doi:10.1038/s41598-024-77151-w)
Supplement: Supplementary file 1 — Supplementary Material 1 [file 41598_2024_77151_MOESM1_ESM.pdf]

Supplementary Materials

Supplementary Results

Supplementary Figure i.

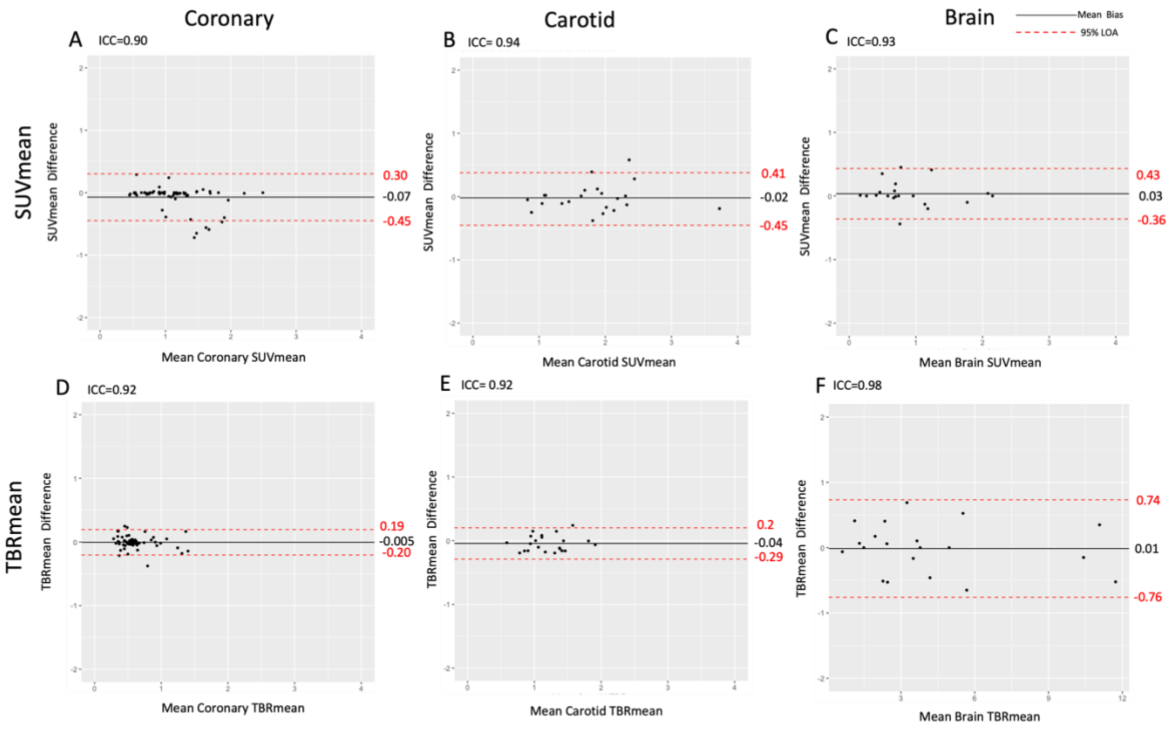

Supplementary Figure i.

Intraobserver levels of Agreement in Quantitative Analysis of ^{18}F-GP1 Uptake.

Figures show mean standardised uptake values (SUV_{mean}; first row A-C), mean target to background ration (TBR_{mean}; second row d-f), for coronary artery (first column), carotid arteries (second column) and brain (third column).

ICC = intraclass correlation coefficient, LOA= Limits of agreement.

Please note the difference in x-axis scale for brain TBR_{mean}.

Supplementary Figure ii.

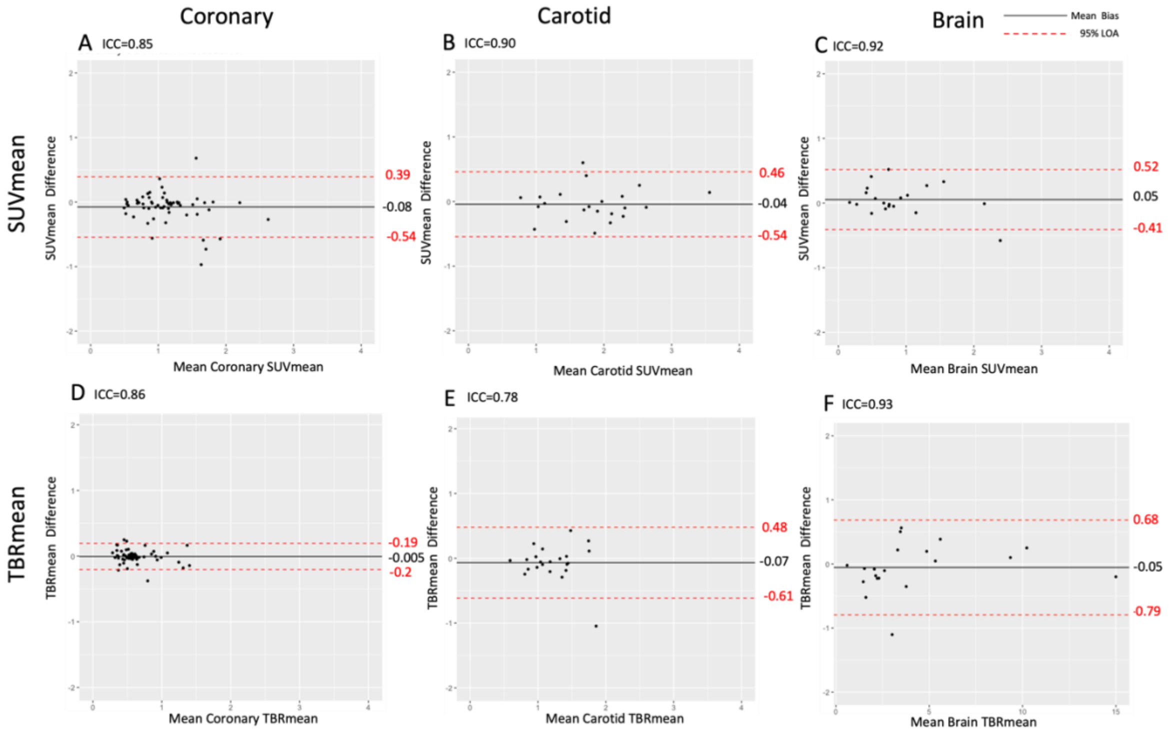

Supplementary Figure ii. Interobserver levels of Agreement in Quantitative Analysis of ^{18}F-GP1 Uptake.

Figures show mean standardised uptake values (SUV_{mean}; first row A-C), mean target to background ration (TBR_{mean}; second row D-F), for coronary artery (first column), carotid arteries (second column) and brain (third column).

ICC = intraclass correlation coefficient, LOA= Limits of agreement.

Please note the difference in x-axis scale for brain TBR_{mean}.
